# Neonatal effect of remifentanil in general anaesthesia for caesarean section: a randomized trial

**DOI:** 10.1186/s12871-015-0020-1

**Published:** 2015-03-26

**Authors:** Pavlina Noskova, Jan Blaha, Hana Bakhouche, Jana Kubatova, Jitka Ulrichova, Patricia Marusicova, Jan Smisek, Antonin Parizek, Ondrej Slanar, Pavel Michalek

**Affiliations:** 1 Department of Anaesthesiology, Resuscitation and Intensive Medicine, 1st Faculty of Medicine, Charles University in Prague and General University Hospital in Prague, U Nemocnice 2, 128 08 Praha 2, Czech Republic; 2 Institute of Pharmacology, 1st Faculty of Medicine, Charles University in Prague and General University Hospital in Prague, Albertov 4, 128 00 Praha 2, Czech Republic; 3 Neonatology, Department of Gynaecology and Obstetrics, 1st Faculty of Medicine, Charles University and General University Hospital in Prague, Apolinarska 18, 128 51 Praha 2, Czech Republic; 4 Department of Gynaecology and Obstetrics, 1st Faculty of Medicine, Charles University and General University Hospital in Prague, Apolinarska 18, 128 51 Praha 2, Czech Republic

**Keywords:** Remifentanil, Neonatal effect, General anaesthesia, Caesarean delivery

## Abstract

**Background:**

Remifentanil has been suggested for its short duration of action to replace standard opioids for induction of general anaesthesia in caesarean section. While the stabilizing effect of remifentanil on maternal circulation has been confirmed, its effect on postnatal adaptation remains unclear, as currently published studies are not powered sufficiently to detect any clinical effect of remifentanil on the newborn.

**Methods:**

Using a double-blinded randomized design, a total of 151 parturients undergoing caesarean delivery under general anaesthesia were randomized into two groups – 76 patients received a bolus of remifentanil prior to induction, while 75 patients were assigned to the control group. Remifentanil 1 μg/kg was administered 30 seconds before the standard induction of general anaesthesia. The primary outcome measure was an assessment of neonatal adaptation using the Apgar score, while secondary outcomes included the need for respiratory support after delivery and differences in umbilical blood gas analysis (Astrup).

**Results:**

The incidence of lower Apgar scores between 0 and 7 was significantly higher in the remifentanil group at one minute (25% vs. 9.3% of newborns, p = 0.017); whilst at five minutes and later no Apgar score differences were observed. There was no difference in the need for moderate (nasal CPAP) or intensive (intubation) respiratory support, but significantly more neonates in the remifentanil group required tactile stimulation for breathing support (21% vs. 7% of newborns, p = 0.017). There was no difference in the parameters from umbilical cord blood gas analysis between the groups.

**Conclusion:**

At a dose of 1 μg/kg, remifentanil prior to induction of general anaesthesia increases the risk of neonatal respiratory depression during first minutes after caesarean delivery but duration of clinical symptoms is short.

**Trial registration:**

ClinicalTrials.gov: NCT01550640.

## Background

Opioids are routinely avoided during induction to general anaesthesia for caesarean section because of the potential for respiratory depression in the neonate [1,2]. On the other hand, insufficient depth of analgesia in parturients until foetal delivery remains a concern for obstetric anaesthetists [3,4]. Therefore, the ultra short-acting μ1-receptor agonist remifentanil has been suggested as a replacement for longer acting opioids in parturients undergoing caesarean delivery [5-11]. Remifentanil rapidly crosses the placenta but simultaneously is quickly eliminated from the neonatal circulation by degradation with nonspecific esterases in plasma and/or redistribution [12-14]. With a half-life of 3–10 minutes, remifentanil should be almost entirely eliminated from foetal circulation by the time of delivery [15]. However, in obstetrics, despite promising pharmacokinetics and pharmacodynamics, remifentanil is currently more frequently used as a systemic alternative to epidural labour analgesia rather than for general anaesthesia for caesarean delivery [16].

While the stabilizing effect of remifentanil on maternal circulation has been clearly and consistently described in several studies, including the systematic review and meta-analysis published by Heesen and colleagues [17], the effect on postnatal adaptation remains unclear, as these studies have not been sufficiently powered. We therefore aimed our study primarily as a comparison of postnatal adaptation of neonates after caesarean delivery in parturients receiving remifentanil bolus of 1 μg/kg prior to induction of general anaesthesia with those having standard opioid-free induction.

## Methods

### Study design

This prospective, randomized, controlled, and double-blinded study was conducted at a tertiary care university hospital with an average of 4,600 births per year, in the period between March 2011 and April 2014.The study was approved by the Ethics Committee of the General University Hospital in Prague (MZ10-UK1LF-Slanar) and registered at the Clinical Trials Database (ClinicalTrials.gov NCT01550640). Study was conducted in accordance with Helsinki Declaration principles. Signed informed consent was obtained from each participant.

Primary objective of the study was to compare newborn postnatal adaptation in parturients undergoing caesarean delivery under general anaesthesia with a remifentanil bolus 1 μg/kg administered prior to the induction of general anaesthesia with those undergoing standard induction. Secondary outcomes were requirements for postoperative respiratory support of neonates and differences in umbilical cord blood gas analysis.

### Study population

Eligible patients were parturients undergoing caesarean delivery under general anaesthesia. Patients were screened and enrolled in the study by an anaesthetist during the pre-anaesthesia visit before caesarean delivery. Inclusion criteria were: age of 18 – 45 years. Exclusion criteria included known allergy to remifentanil, multiple pregnancy, gestational age below the 35th week, estimated weight of foetus below 2500 grams, severe foetal hypoxia, severe maternal hypotension, and other serious foetal or maternal conditions. Discontinuation criterion was difficult foetal delivery defined as uterine incision-to-delivery interval >3 min.

### Study interventions

Parturients were randomly assigned to two study groups - remifentanil (RMF) or standard (STD). Treatment allocation was performed using online randomization generator (www.randomization.com). Each patient was allocated before entering the operating room. The randomization was kept blinded for the patient, surgeon, and neonatologist. Patients in the RMF group received a bolus of remifentanil 1 μg/kg 30 seconds prior to induction with thiopentone; while all other preoperative, anaesthetic, obstetric and postoperative procedures, were identical for both groups.

### General anaesthesia

Standard departmental protocol for caesarean delivery under general anaesthesia was used with metoclopramide and ranitidine administered orally for aspiration prevention. After preoxygenation, general anaesthesia was induced using i.v. thiopentone 5 mg/kg followed by succinylcholine 1.25 mg/kg, and, until delivery, maintained with sevoflurane at an expired concentration of 0.5-0.7% in a 50% gas mixture of nitrous oxide/oxygen. After ligation of the umbilical cord, sufentanil 0.5 μg/kg and atracurium 0.35 mg/kg were administered for analgesia and muscle relaxation and sevoflurane was increased to 1%.

### Obstetric management

Pfannenstiel supra-cervical laparotomy and Geppert uterotomy were used in all cases. Oxytocin 5 IU as a bolus was administered intravenously, diluted in 20 ml of saline after removal of the placenta.

### Monitoring and evaluation

Intraoperative monitoring was performed using Datex Ohmeda S/5 TM Compact Anaesthesia Monitor (Datex-Ohmeda Inc., USA) according to general standards for patient monitoring during general anaesthesia. Time 0 was determined as the time of remifentanil administration in the RMF group or time 30 sec prior to thiopentone administration in the STD group. The following parameters were monitored; systolic and diastolic non-invasive blood pressure (NIBPsyst; NIBPdiast), mean blood pressure (MAP), heart rate (HR), electrocardiography (ECG) with ST segment analysis (lead II), pulse oximetry (SpO_2_), capnography (EtCO_2_), % of oxygen and volatile anaesthetic, and ventilation parameters (minute ventilation, tidal volume, respiratory rate and maximal inspiratory pressure). To evaluate the depth of anaesthesia, continuous bispectral index analysis (BIS) was used.

Evaluation of newborn adaptation was performed using Apgar scores at 1, 5 and 10 minutes, simultaneously with arterial and venous umbilical cord blood gas analysis and clinical examination. Clinical assessment was undertaken by an experienced neonatologist and acid/base balance status was evaluated directly in the delivery room with ABL 90 Flex (Radiometer Medical, Denmark) blood gas analyser.

### Statistical analysis

The sample size of 150 parturients was calculated to detect an overall twofold difference between the groups in the primary outcome (Apgar scores 0–7) with a two-sided 5% significance level and a power of 80%. For sample size calculation, we used data from long term departmental baseline statistics, where Apgar score of 0–7 was observed in 9.5% of caesarean sections under general anaesthesia. A 36-month inclusion period was anticipated to recruit this number of patients.

Statistical analysis was performed with the STATISTICA 10 software (StatSoft, Czech Republic). All data was tested for normality using Kolmogorov-Smirnov test prior to final analysis. Numerical data from both groups was compared using Student’s t-test or Mann–Whitney Rank Sum Test, as appropriate. For categorical variables the Fisher’s exact test and chi-square test were used. All statistical tests were performed two-tailed and values of P < 0.05 were considered statistically significant.

## Results

**Figure 1:**
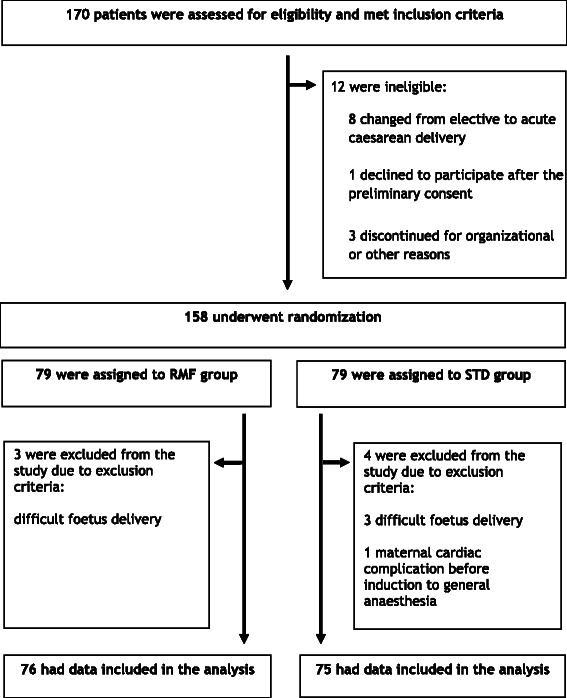
**Assessment, randomization, and follow-up of the study patients.**

A total of 151 parturients were included in the study, 76 patients were allocated to the remifentanil group while another 75 were assigned to the standard group. All participants were in physical status class I-II according to the American Society of Anesthesiologists. Demographic characteristics of parturients are expressed in Table 1. Figure 1 represents study flow diagram.

**Table 1:** **Characteristics of parturients and newborns**

	Remifentanil group (n = 76)	Standard group (n = 75)	*P*
Age (years)	33.1 ± 5.1	32.3 ± 5.3	*0.362*
Weight prior to pregnancy (kg)	68.1 ± 13.1	69.8 ± 13.4	*0.473*
Actual weight (kg)	82.0 ± 13.4	81.4 ± 14.3	*0.813*
Height (cm)	168.9 ± 6.8	167.6 ± 5.8	*0.233*
BMI (kg.m^−2^)	24.0 ± 4.4	24.9 ± 5.0	*0.254*
***Medical concomitant diseases***			
Hypertension	6 (8%)	6 (8%)	*0.981*
Asthma	4 (5%)	3 (4%)	*0.712*
Thyreopathy	3 (4%)	3 (4%)	*0.987*
Diabetes	3 (4%)	2 (3%)	*0.660*
Placenta praevia centralis	3 (4%)	3 (4%)	*0.987*
Other	11 (14%)	13 (17%)	*0.796*
***Newborns***			
Weight of newborns (g)	3162.9 ± 467	3122.5 ± 618	*0.334*
Gestational age (weeks)	38.6 ± 1.1	38.9 ± 1.4	*0.541*

Data are presented as mean ± standard deviation or n (%).

BMI = Body Mass Index.

The most frequent reason for employment of general anaesthesia in this study was refusal of regional anaesthesia by the parturient (67 vs. 68 patients). In only 9 (RMF group) and 7 (STD group) cases, respectively, the choice of general anaesthesia was based on medical considerations - placenta praevia centralis (6 pts.), thrombocytopenia (3 pts.), psychosis (3 pts.), tokophobia (2 pts.), history of spinal trauma (1 pt.) and hypersensitive tetany (1 pt.).

Anaesthesia and surgical baseline management characteristics including delivery time (induction to delivery interval) are shown in Table 2. No intra- or postoperative major complications were recorded during the study.

**Table 2:** **Data related to general anaesthesia**

	Remifentanil group (n = 76)	Standard group (n = 75)	*P*
**Anaesthesia prior foetal delivery**			
Thiopentone bolus (mg)	398 ± 64	401 ± 68	*0.745*
Sevoflurane (%)	0.57 ± 0.2	0.58 ± 0.3	*0.586*
SatO_2_ (%)	98.4 ± 1.7	97.9 ± 2.3	*0.219*
ETCO_2_ (kPa)	4.5 ± 0.5	4.4 ± 0.4	*0.529*
Induction-to-delivery interval (min)	4.0 ± 1.4	3.9 ± 1.2	*0.884*
Duration of surgery (min)	43 ± 9.2	41 ± 9.4	*0.724*
Estimated blood loss (ml)	594 ± 196	576 ± 148	*0.648*
**BIS** (%)			
Baseline	95.4 ± 4.3	96.2 ± 4.2	*0.360*
Tracheal intubation	52.4 ± 16	52.2 ± 12.9	*0.930*
Delivery	57.7 ± 13.7	55.7 ± 12.2	*0.401*
At 10 min after induction	58.3 ± 10.5	55.8 ± 12.3	*0.231*

Data are presented as mean ± standard deviation. Sevoflurane = mean end expiratory concentration of sevoflurane from induction to delivery; SatO_2_ = mean oxygen saturation from induction to delivery; ETCO_2_ = mean end tidal capnography from induction to delivery; BIS = bispectral index analysis. Baseline means time before induction to general anaesthesia.

### Neonatal outcome

**Figure 2:**
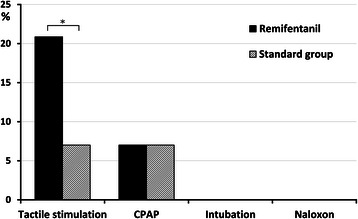
**Bar graph showing the need for management of newborn respiratory depression.** The bar graph plots the percentage of newborns requiring each level of respiratory stimulation or support (n = 76 in the remifentanil group and n = 75 in standard group). The black column represents the remifentanil group; the cross-hatched column the control group. *P = 0.017. CPAP = continuous positive airway pressure ventilation.

Table 3 and Figure 2 show the characteristics of neonatal postnatal adaptation – Apgar scores and the need for respiratory support. We noted a higher incidence of low Apgar scores between 0 and 7 in the remifentanil group at the first minute (19 vs. 7 newborns, p = 0.017), but after the fifth minute no difference between the groups was observed. No difference between the study groups was found in requirement for moderate or intensive respiratory support following delivery (p = 0.983). Temporary CPAP (continuous positive airway pressure) ventilation was employed in 10 newborns (5 in each group), but none required intubation or admission to a neonatal intensive care unit. There was however, a significant difference in the need for mild breathing support using tactile stimulation during the first 5 minutes (16 vs. 5 newborns, p = 0.017) (Table 3). No newborn required administration of an μ1-opioid receptor antagonist.

**Table 3:** **Neonatal outcome**

	Remifentanil group (n = 76)	Standard group (n = 75)	P value
**Apgar score**			
1-minute	8.1 ± 2.0	8.9 ± 1.4	*0.005*
5-minute	9.2 ± 1.1	9.6 ± 0.8	*0.022*
10-minute	9.8 ± 0.5	9.8 ± 0.4	*0.198*
**Apgar score 0 -7**			
1-minute	19 (25.0%)	7 (9.3%)	*0.017*
5-minute	5 (6.6%)	2 (2.7%)	*0.442*
10-minute	0 (0%)	0 (0%)	*-*
**Need for respiratory support**			
Tactile stimulation	16 (21.1%)	5 (6.7%)	*0.017*
CPAP	5 (6.6%)	5 (6.7%)	*0.983*
Mechanical ventilation	0 (0%)	0 (0%)	*-*
Administration of naloxone	0 (0%)	0 (0%)	*-*

Data are presented as mean ± standard deviation or n (%).

CPAP = continuous positive airway pressure ventilation.

Table 4 shows umbilical cord arterial and venous blood gas analysis. No difference was observed for any parameter including number of pH values <7.2; such a value was recorded only once (7.18, in the remifentanil group).

**Table 4:** **Umbilical cord blood gas analysis**

	Remifentanil group (n = 76)	Standard group (n = 75)	P value
**Arterial**			
pH	7.3 ± 0.0	7.3 ± 0.0	*0.210*
pCO_2_ (kPa)	6.7 ± 0.7	6.6 ± 0.7	*0.450*
HCO_3_-act (mmol/l)	25.0 ± 1.9	24.9 ± 1.9	*0.786*
HCO_3_-std (mmol/l)	22.2 ± 1.9	22.0 ± 1.3	*0.567*
BE (mmol/l)	−1.4 ± 1.7	−1.2 ± 1.7	*0.645*
pO_2_ (kPa)	2.8 ± 0.6	2.6 ± 0.6	*0.189*
satO_2_ (%)	41.9 ± 14.6	38.0 ± 13.6	*0.137*
**Venous**			
pH	7.4 ± 0.0	7.3 ± 0.0	*0.203*
pCO_2_ (kPa)	5.8 ± 0.5	5.8 ± 0.6	*0.721*
HCO_3_-act (mmol/l)	23.5 ± 1.6	23.4 ± 1.9	*0.848*
HCO_3_-std (mmol/l)	22.4 ± 1.1	22.2 ± 1.4	*0.421*
BE (mmol/l)	−1.7 ± 1.7	−1.8 ± 1.7	*0.801*
pO_2_ (kPa)	4.6 ± 0.8	4.3 ± 1.1	*0.157*
satO_2_ (%)	72.6 ± 10.4	68.3 ± 14.4	*0.069*

Data are presented as mean ± standard deviation.

#### Maternal haemodynamics

The study groups were equal in basal haemodynamic parameters (blood pressure and heart rate at time 0), but they varied significantly at the time of intubation (both blood pressure and heart rate) and delivery (heart rate). The basal haemodynamic profiles are shown in Table 5.

**Table 5:** **Maternal haemodynamic profiles**

	Remifentanil group (n = 76)	Standard group (n = 75)	*P*
**Systolic blood pressure** (mmHg)			
Baseline	140.8 ± 16.2	140.6 ± 18.0	*0.948*
At intubation	145.4 ± 23.5	164.1 ± 26.4	<0.0001
At delivery	146.6 ± 22.5	149.9 ± 25.4	*0.444*
At 10 min after induction	125.7 ± 17.3	122.5 ± 17.0	*0.283*
**Heart rate** (bpm)			
Baseline	89.4 ± 12.8	93.1 ± 16.9	*0.187*
At intubation	91.8 ± 16.6	109.7 ± 17.2	<0.0001
At delivery	88.9 ± 13.7	101.3 ± 20.1	<0.001
At 10 min after induction	73.0 ± 16.3	76.6 ± 14.7	*0.204*

Data are presented as mean ± standard deviation. Baseline means time before induction to general anaesthesia.

#### Depth of anaesthesia

No difference was found between the groups after induction of general anaesthesia in depth of anaesthesia (BIS), ST-analysis, or respiratory-ventilation parameters (SatO_2_, EtCO_2_). The profiles of BIS values are shown in Table 2.

## Discussion

Our study is not the first one to evaluate the effect of remifentanil in parturients undergoing caesarean delivery under general anaesthesia. However, to our knowledge, it is the only one adequately powered to assess the effect on the incidence of respiratory depression during postnatal adaptation of newborns. Moreover, the other important aspect of our study is its sample size. With 151 patients participating in the study and 76 in the remifentanil group, this is the largest remifentanil assessment in obstetric patients undergoing caesarean delivery. The size of our homogenous study group should be highlighted especially in comparison to the meta-analysis conducted and published in 2013 by a German-Belgian team with the inclusion of 186 patients from 5 randomized clinical trials [17].

While published studies agree on the positive effect of remifentanil on reducing adverse haemodynamic response to intubation and surgery during caesarean delivery, they vary in terms of its effect on neonatal adaptation. Yoo [18] and Ngan Kee [15] used an identical bolus of remifentanil - 1 μg/kg. They highlighted, similarly to our study, a risk of transient, neonatal respiratory depression. In contrast, Bouattour and colleagues did not demonstrate any attenuation of neonatal adaptation after administration of 0.5 μg/kg of remifentanil [19]. More worryingly, 14.3% of newborns in another study had to be intubated for respiratory support, following a pre-induction dose of remifentanil 0.5 μg/kg [20]. The determination of a remifentanil “safe dose”, not causing adverse effect on neonate, was not even unravelled by the aforementioned meta-analysis performed by Heesen et al. [17].

In our study, we observed a significantly higher incidence of moderate to severe aggravation of neonatal adaptation (Apgar scores 0–7) after remifentanil administration (p =0.017). However, in all cases, neonatal respiratory depression lasted only for several minutes because lower Apgar scores were observed at the 1st minute only. The Apgar scores were similar at 5 minutes, which is a more crucial time for subsequent neonatal care and/or eventual transfer to neonatal intensive care unit. In total, 28% of neonates in the remifentanil group required some form of respiratory support after delivery, however they mostly responded only to tactile stimulation.

The observed incidence of short-time lasting, deteriorated postpartum neonatal adaptation was quite high in the remifentanil group, although its clinical significance and severity are not clear. A hypothetical explanation of different results in published studies could be the existence of genetic polymorphism of the placenta transporting system glycoprotein P (multidrug resistance gene 1, MDR1) and polymorphism of μ-receptors and then divergent transfer of remifentanil into the foetal circulation [21,22]. Another reason for relatively higher incidence of respiratory depression in our study may also be shorter time of foetal delivery (4.0 min induction-to-delivery time) compared to some other centres [20,23,24]. We may hypothesize that remifentanil with its half-life of 3–10 minutes was not completely eliminated at the time of delivery. This could explain why Yoo et al. [24] found no significantly higher depression rate, even with a dose of 1.25 μg/kg. It should be noted that they reported interval to delivery approximately 10 minutes, which is potentially longer than expected effect of remifentanil.

It should be also highlighted that postnatal adaptation of the newborn is affected by previous intrauterine foetal condition, concurrent administration of thiopentone and volatile anaesthetics, as well as by induction-to-delivery interval and technique of caesarean section. Therefore we excluded parturients with expected alteration of neonatal respiratory function (immaturity of foetus or in utero pathologies). In order to decrease bias of the study, we also excluded females with multiple pregnancy (risk of low birth weight and prematurity) and newborns following difficult delivery with uterine incision-to-delivery interval more than 3 min [25-27]. Nevertheless, we observed 14% incidence of moderate respiratory depression even in the standard group, with 5 newborns requiring the use of CPAP. Specific reasons for this depression remain unclear even after detailed analysis of individual cases (with no noted significant differences in delivery interval, gestational age or weight, blood gas analysis results or maternal demographic, respiratory or haemodynamic parameters).

In contrast to other studies, we did not find any difference in the umbilical cord blood gas analysis parameters, including Base Excess reported in Heesen’s meta-analysis [17]. Acid/base balance parameters in umbilical cord blood correspond with the status of foetal oxygenation at the time of delivery. We detected only one newborn in the remifentanil group presenting with pH <7.2, which may be associated with increased neonatal morbidity [28]. Therefore, our assessment of remifentanil effect on neonatal adaptation should not have been affected by concurrent foetal intrauterine hypoxia.

We are aware of limitations of our study. Although the adaptation of each newborn was evaluated by experienced neonatologist, requirements to stimulate their breathing by tactile stimulation might still be biased by inter-individual differences. Another limitation may be exclusion of deliveries indicated for acute foetal hypoxia. General anaesthesia is most commonly administered in such cases, where suppression of stress response to tracheal intubation and surgical stimuli would be theoretically desirable.

Forming a consensus with other published trials, we also recorded the positive effect of remifentanil on the suppression of cardiovascular stress response to tracheal intubation and surgery. A dose of remifentanil 1 μg/kg given 30 seconds prior to induction of general anaesthesia effectively reduced a rise in both blood pressure and heart rate until delivery of foetus. Whereas the haemodynamic monitoring wasn’t the main aim of this study, it was not blinded for anaesthesiologist. Therefore its assessment might be burdened by a potential bias, albeit most likely only an insignificant. The haemodynamic profile herein is given primarily within an overall description of intraoperative course of our study.

Unlike its stabilizing effect on the cardiovascular system, the dose of remifentanil 1 μg/kg did not affect the depth of anaesthesia. BIS values were below 60 in both groups throughout the study. This value has been accepted as a threshold which should decrease the incidence of awareness [29,30]. Similar results have been reported in other studies [18]. It is assumed that the majority of hypnotic effect during induction is achieved by the initial dose of thiopentone potentiated by anaesthetic gases, while remifentanil in this dose has only small effect on depth of anaesthesia [31].

## Conclusion

We demonstrated that a pre-induction dose of remifentanil 1 μg/kg is associated with a relatively high risk of neonatal respiratory depression. However, this attenuation was present only in the first five minutes after delivery and its clinical significance seems to be rather mild. Remifentanil is a suitable choice of co-induction agent in parturients who could be prone to excessive hypertension and tachycardia during induction to anaesthesia. In these cases, careful postnatal care must be applied if respiratory depression is observed.

## Abbreviations

BIS: Bispectral index analysis
BMI: Body Mass Index
BP: Blood pressure
CPAP: Continuous positive airway pressure
ECG: Electrocardiography
EtCO_2_: End-tidal capnography
IU: International unit
HR: Heart rate
MAP: Mean blood pressure
NIBPsyst: Systolic non-invasive blood pressure
NIBPdiast: Diastolic non-invasive blood pressure
Sat O_2_: Saturation
SD: Standard deviation
